# Candidate Genes of Gastrointestinal Nematode Resistance Traits in Sheep: A Systematic Review of GWASs and Gene Prioritization Analysis

**DOI:** 10.3390/genes16101151

**Published:** 2025-09-28

**Authors:** Zhirou Zhang, Gang Liu, Deji Xu, Yueqi Ma, Xianlong Wang, Yong Wang, Lei Hou, Jiaqing Hu, Jianmin Wang, Tianle Chao

**Affiliations:** 1 Shandong Provincial Key Laboratory for Livestock Germplasm Innovation & Utilization, College of Animal Science and Technology, Shandong Agricultural University, Tai’an 271018, China; zzrzhang0711@126.com (Z.Z.); 19306491839@163.com (D.X.); 13704187907@126.com (Y.M.); jqh0609@sdau.edu.cn (J.H.); wangjm@sdau.edu.cn (J.W.); 2 Key Laboratory of Efficient Utilization of Non-Grain Feed Resources (Co-Construction by Ministry and Province), Ministry of Agriculture and Rural Affairs, College of Animal Science and Technology, Shandong Agricultural University, Tai’an 271018, China; 3 Shandong Provincial Animal Husbandry Station (Shandong Province Breeding Livestock and Poultry Quality Measuring Station), Jinan 250100, China; shannong@163.com; 4 Jinan Animal Disease Prevention and Control Center, Jinan 250022, China; wxl19750905@163.com; 5 Jinan Animal Husbandry Technology Extension Station, Jinan 250300, China; 386891170@163.com; 6 Tai’an Animal Husbandry and Veterinary Service Center, Tai’an 271018, China; hllhyy@163.com

**Keywords:** sheep, gastrointestinal nematodes, parasite resistance, genome-wide association study, GWAS, livestock breeding, candidate gene, genetic markers

## Abstract

**Background/Objectives:** Gastrointestinal nematode infections represent a major constraint to sheep production globally, with widespread drug resistance requiring alternative control strategies. **Methods:** This systematic review combined genome-wide association study findings to understand the genetic basis underlying parasite resistance traits in sheep. Following PRISMA guidelines, we identified 22 studies including 28,033 samples from 32 breeds across 11 countries, extracting 1580 candidate genes associated with resistance traits, including fecal egg count, packed cell volume, and immunoglobulin levels. Gene prioritization analysis using ToppGene identified 75 high-confidence candidate genes. **Results**: Functional enrichment analysis revealed significant involvement of the JAK-STAT signaling pathway, inflammatory response processes, and immune-related biological functions. Protein–protein interaction network analysis identified nine key hub genes: TNF, STAT3, STAT5A, PDGFB, ADRB2, MAPT, ITGB3, SMO, and GH1. The JAK-STAT pathway emerged as particularly important, with multiple core genes involved in cytokine signaling and immune cell development. These findings demonstrate that parasite resistance involves complex interactions between inflammatory responses, immune signaling networks, and metabolic processes. **Conclusions**: This comprehensive genetic framework provides essential insights for developing genomic selection strategies and marker-assisted breeding programs to enhance natural parasite resistance in sheep, offering a sustainable approach to reducing drug dependence and improving animal welfare in global sheep production systems.

## 1. Introduction

Sheep serve as important livestock animals in global agricultural economies, playing a central role by providing meat, milk, wool and other products for human use. However, gastrointestinal nematode (GIN) infections have become one of the most significant factors limiting the sustainable development of the global sheep industry [1,2,3]. These parasites primarily inhabit the small intestine and abomasum of sheep, causing symptoms including reduced appetite, weight loss, diarrhea, hypoproteinemia, and anemia. In severe cases, these infections can lead to death, resulting in substantial economic losses for the livestock industry [4,5].

Traditional GIN control strategies have mainly relied on anthelmintic drug treatments. However, prolonged, frequent, and improper use of anthelmintic drugs has led to rapid development and widespread distribution of parasite drug resistance, a problem that has become increasingly serious worldwide [6,7]. Research shows that many sheep farms in several Southern Hemisphere countries now face multiple drug resistance issues, seriously threatening the effectiveness of traditional pharmaceutical control methods [8]. The spread of anthelmintic resistance not only reduces treatment effectiveness but also creates potential risks to food safety and the environment, making strategies that rely solely on chemical control unsustainable [9].

Given this situation, developing sustainable and environmentally friendly alternative GIN control strategies has become increasingly urgent. Among these approaches, breeding sheep with enhanced parasite resistance through genetic selection is considered one of the most promising solutions [10,11]. This method can reduce dependence on anthelmintic drugs, decrease the selection pressure for resistance development, and provide long-term, sustainable benefits for livestock production [12,13].

Sheep show significant breed-to-breed and individual-to-individual genetic variation in resistance and tolerance to GIN infections, providing a genetic foundation for improving resistance through selective breeding [14,15]. For example, indigenous breeds such as Djallonké sheep from Africa, Red Maasai sheep, and Barbados Black Belly sheep from the Caribbean region demonstrate stronger natural parasite resistance compared to European improved breeds [16]. Recent meta-analyses indicate that parasite resistance is a moderately heritable complex quantitative trait, with heritability estimates for FEC ranging from 0.01 to 0.65 across different sheep breeds globally, suggesting good selection potential for this trait [17].

Currently, assessment of parasite resistance traits in sheep relies mainly on phenotypic indicators including fecal egg count (FEC), packed cell volume (PCV), FAMACHA^©^ eyelid scoring, and immunoglobulin A (IgA) levels [18,19]. However, these phenotypic measurements are typically time-consuming, expensive, and labor-intensive, and are easily affected by environmental factors, animal physiological status, measurement timing, and other variables. These limitations significantly restrict the application of traditional breeding methods in large-scale commercial production.

The rapid development of genomic technologies, particularly the emergence of genome-wide association studies (GWASs), has provided new opportunities for revealing the genetic architecture of complex traits [20,21]. GWASs can systematically scan entire genomes to identify single nucleotide polymorphisms (SNPs) and genomic regions significantly associated with target traits, and can further annotate functional candidate genes [22,23]. In recent years, multiple GWASs based on different sheep breeds have identified numerous candidate genes related to parasite resistance traits, which are mainly involved in biological processes such as immune response, inflammatory response, and cytokine signal transduction [24,25]. Case–control GWASs based on estimated breeding values show that genomic data can effectively distinguish animals with the highest and lowest genetic resistance to gastrointestinal nematode infections [26]. Recent research indicates that incorporating genomic data into genetic prediction models can significantly improve breeding value prediction accuracy, providing strong support for the application of genomic selection in sheep parasite resistance breeding [27]. Additionally, studies have found that traditionally managed indigenous sheep have evolved various strategies to enhance GIN resistance through long-term natural selection processes [28].

Although individual GWASs have provided valuable information for understanding the genetic basis of parasite resistance traits in sheep, differences between studies in breeds, environmental conditions, sample sizes, and analytical methods have resulted in some heterogeneity and inconsistency in results. Moreover, many candidate genes identified in studies lack functional validation, and their specific roles in parasite resistance mechanisms remain unclear. Therefore, there is an urgent need to integrate existing research results through systematic reviews and meta-analyses to identify the most reliable candidate genes and prioritize and functionally annotate them.

This study aims to conduct a systematic literature review and gene priority analysis to comprehensively examine progress in GWAS-based sheep parasite resistance candidate gene research, integrate genetic discoveries from different studies, identify high-confidence functional candidate genes, and explore the related biological pathways and molecular mechanisms in detail. The results will provide important insights for understanding the genetic architecture of sheep parasite resistance traits, offer scientific evidence for genomic selection breeding and molecular marker-assisted selection, and ultimately promote the development of parasite-resistant sheep breeds and sustainable livestock production.

## 2. Materials and Methods

### 2.1. Systematic Review Search Strategy

**Figure 1:**
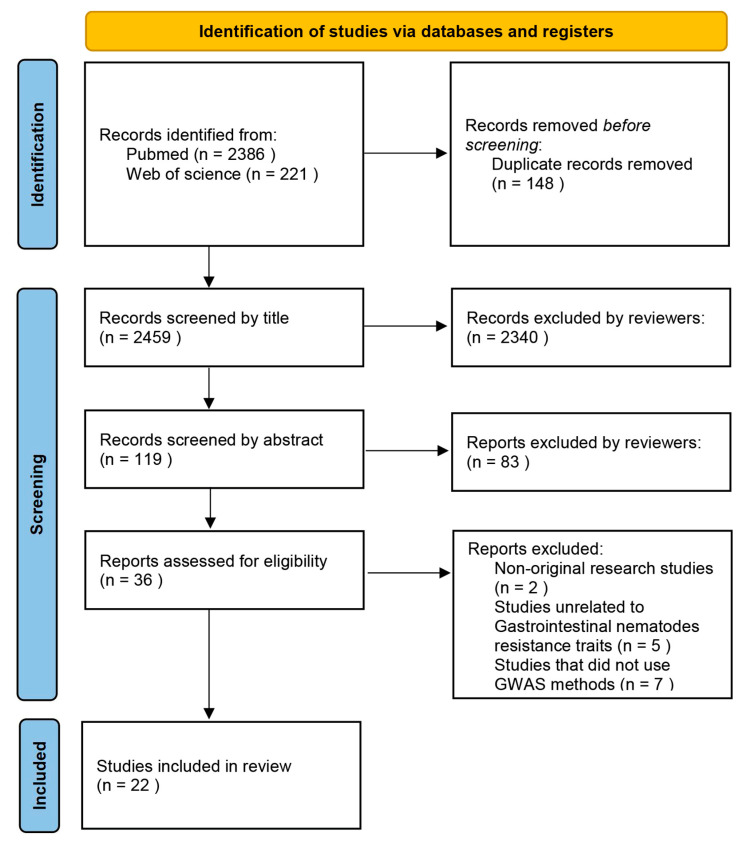
Flowchart of the literature search strategy and study selection.

This systematic review followed a pre-specified research protocol and the Preferred Reporting Items for Systematic Reviews and Meta-Analyses (PRISMA) statement [29], as shown in Figure 1. Online databases and conference proceedings were searched to identify the relevant literature. The online databases searched included NCBI-PubMed and Web of Science from database inception to 20 August 2025, with papers written in English only.

The search terms used for the NCBI-PubMed database were “((sheep[MeSH Terms] OR ovine[Title/Abstract] OR lamb[Title/Abstract] OR “Ovis aries”[Title/Abstract]) AND (resistance[Title/Abstract] OR resistant[Title/Abstract] OR susceptibility[Title/Abstract] OR susceptible[Title/Abstract] OR tolerance[Title/Abstract] OR tolerant[Title/Abstract]) OR (nematode*[Title/Abstract] OR parasite*[Title/Abstract] OR helminth*[Title/Abstract] OR worm*[Title/Abstract] OR roundworm*[Title/Abstract] OR “gastrointestinal parasite*”[Title/Abstract] OR “intestinal parasite*”[Title/Abstract] OR Haemonchus[Title/Abstract] OR Teladorsagia[Title/Abstract] OR Trichostrongylus[Title/Abstract] OR Cooperia[Title/Abstract]) AND (“genome-wide association study”[MeSH Terms] OR “genome-wide association stud*”[Title/Abstract] OR GWAS[Title/Abstract] OR “genetic association*”[Title/Abstract] OR “association mapping”[Title/Abstract] OR “quantitative trait loc*”[Title/Abstract] OR QTL[Title/Abstract] OR “single nucleotide polymorphism*”[Title/Abstract] OR SNP[Title/Abstract] OR “genomic selection”[Title/Abstract])) AND (“01 January 1900”[Date-Publication]: “20 August 2025”[Date-Publication])”. This yielded 2386 initial records.

The search terms for Web of Science were “TS = ((sheep OR ovine OR lamb OR “Ovis aries”) AND (resistance OR resistant OR susceptibility OR susceptible OR tolerance OR tolerant) AND (nematode* OR parasite* OR helminth* OR worm* OR roundworm* OR “gastrointestinal parasite*” OR “intestinal parasite*” OR Haemonchus OR Teladorsagia OR Trichostrongylus OR Cooperia) AND (“genome-wide association stud*” OR GWAS OR “genetic association*” OR “association mapping” OR “quantitative trait loc*” OR QTL OR “single nucleotide polymorphism*” OR SNP OR “genomic selection”))”, with a publication date between 1 January 1900 and 20 August 2025. This yielded 221 initial records.

### 2.2. Literature Inclusion and Selection Process

Each abstract was independently screened by Zhang Zhirou and Chao Tianle. Abstract selection was based on the following criteria: (1) original research article; (2) focus species: sheep; and (3) mention of GWAS-identified mutation sites such as SNPs, indels, copy number variations and haplotypes, or candidate genes. During full-text review, articles were excluded if (1) GWAS technology was not used for analysis; or (2) defined phenotypes did not include gastrointestinal nematode resistance-related traits. Any disagreements between the two reviewers (Zhang Zhirou and Chao Tianle) regarding abstract or full-text inclusion were resolved through discussion and consensus. Further screening of included articles was conducted to search for relevant references, with citation checking performed. For each included full-text study, Microsoft Excel was used to organize the following data; data extraction from eligible full-text articles was performed independently by two reviewers (Zhang Zhirou and Chao Tianle). The extraction form captured the author, year, study design, breed(s), sample size, geographic location, phenotype, platform used, SNP density, QC parameters, population structure control, multiple testing correction, significant SNPs/windows, chromosomal locations, and *p*-values. Each included study was independently extracted by both reviewers, with extractors blinded to each other’s results during the initial phase. Extraction results were compared and disagreements were discussed between the two extractors; persistent disagreements were resolved by consultation with a third reviewer (Liu Gang).

The quality assessment for included GWAS research was performed as in Table 1. Studies with a total score of 10 or higher were classified as high-quality criteria, 7–9 were classified as middle-quality criteria, and 0–6 were classified as low-quality criteria.

### 2.3. Reannotation of Mutation Sites and Extraction of Candidate Genes

For studies that provided specific information on mutation sites, we defined gene-centered windows as gene coordinates ± 100 kb, and used the RGD (Ruminant Genome Database) LiftOver tool to standardize all genomic coordinates to the sheep reference genome ARS-UI_Ramb_v2.0 [30]. The extraction of candidate genes used Ensembl biomart with reference genome version v114 Ramb_v2.0. Among them, candidate genes derived from high-quality criteria studies are classified as highly reliable, while candidate genes from middle-quality criteria studies are classified as moderately reliable. For studies that did not provide specific locus information but provided genomic window information, we directly converted the genomic window regions using the RGD (Ruminant Genome Database) LiftOver tool and extracted candidate genes, but all candidate genes obtained in this way were identified as moderately reliable. For studies where information about loci or gene windows cannot be extracted, the reported candidate genes are directly adopted as low reliable.

### 2.4. Gene Prioritization Analysis

The Sheep QTLdb database [31] was searched using the following terms sequentially: “Parasite resistance”, “Nematode egg count”, “Fecal egg count”, “Worm burden”, “Haemonchus resistance”, “Worm count” and “fecal oocyst count”. The genes retrieved from this search were compiled as “trained genes”. Candidate genes from the literature included in this study were compiled and deduplicated to serve as “test genes”.

Gene prioritization analysis was performed using the online analysis tool ToppGene [32] by inputting “trained genes” and “test genes” with default parameters. Before analysis, genes in “test genes” that overlapped with “trained genes” were removed. Multivariate analyses were performed using functional information shared between test and trained gene sets derived from various sources, including GO for molecular function (MF), GO for biological process (BP), and GO for cellular component (CC); human and mouse phenotypes; pathways; PubMed publications; and diseases. The FDR was calculated based on the overall p-values obtained from the analysis [33], and candidate genes were filtered using FDR ≤ 0.05 as the threshold.

### 2.5. Gene Functional Enrichment Analysis

g: Profiler (Version updated in 16 June 2025) [34] was used to perform GO terms and KEGG pathway enrichment analyses, with an FDR < 0.05 as the threshold.

## 3. Results

### 3.1. Systematic Review

This review evaluated 2459 studies, with 2340 excluded through title screening and an additional 83 excluded through abstract review. Among the remaining 36 studies, 2 were excluded as reviews, 5 were excluded because the parasite resistance studied targeted coccidia, tapeworms, ticks, and other parasites, and 7 were excluded for not using GWAS strategies. A total of 22 studies met the inclusion criteria (Table S1). The PRISMA flow diagram details the step-by-step inclusion and exclusion process, as shown in Figure 1. Study data originated from 11 countries, with Brazil contributing the most studies (6, 27.3%), followed by the United States (4, 18.2%). Experimental animal samples came from 33 breeds/populations, with a total of 28,033 samples included.

Among the 22 included studies (Table S1), 20 used SNP chip platforms exclusively for genotyping, 1 study used a combined imputation strategy involving 50 K chips, 600 K chips, and whole-genome sequencing, and 1 study used targeted sequencing. Twenty-two studies performed association analysis between SNPs and gastrointestinal nematode resistance phenotypes, with 20 of these specifying call rate thresholds and MAF thresholds, 13 specifying multiple correction methods, and 12 specifying Hardy–Weinberg thresholds. Only one study used CNVs as association markers. During the annotation process of marker loci, gene windows, and candidate genes, nine studies used Oar v3.1 as the reference genome, five studies used Oar v4.0, two studies used Ramb v2.0, one used Ramb v1.0, and one study each used Oar v1.0, Oar v2.0 and HuRef assembly v1.0, respectively, while three studies did not specify reference genome versions. We extracted candidate genes related to gastrointestinal nematode resistance phenotypes, including the FEC, WEC, FAMACHA, PCV, TPP, IgA, LW, Haemonchus contortus resistance ranking score, and FEC-EBV, based on the significant markers and genomic window regions reported in the included studies, and used them for subsequent analysis.

Quality assessment revealed substantial heterogeneity in methodological rigor across included studies. A total of 11 studies met high-quality criteria, while other 11 studies met middle-quality criteria, which could have significant methodological limitations (Table S1 and Table 2).

### 3.2. Variant Annotation and Candidate Gene Extraction

In the 11 high-quality criteria studies, a total of 10 provided detailed site information available for extraction, and 3 studies provided genomic window information. A total of 144 SNP sites (Table S2) and 40 genomic windows (Table S3) were successfully extracted and converted according to Ramb v2.0. In the 11 middle-quality criteria studies, 3 studies provided detailed site information available for extraction, 4 provided gene window information, and 5 studies did not provide extractable site or gene window information.

A total of 1580 non-redundant genes were successfully extracted, among which 486 were high-confidence candidate genes, 1118 were middle-confidence candidate genes, and 24 were overlapping between high-confidence and medium-confidence genes (Table S4). Among all 486 high-confidence genes related to the three types of traits, only 1 gene (U6) overlaps between the candidate genes for parasitological traits and immunological traits; no other candidate genes intersect between different traits (Table S4).

Among the 85 high-confidence candidate genes and the 530 middle-confidence candidate genes for immunological traits, only the two genes TRPC4 and U6 are repeated. Among the 385 high-confidence candidate genes and the 349 middle-confidence candidate genes for parasitological traits, only 6 genes (ADGRB3, NUP88, RABEP1, RPAIN, U6, ENSOARG00020035905) are repeated. No overlapping genes were found between physiological traits.

### 3.3. Gene Prioritization Analysis Results

After extraction and deduplication, a total of 1580 candidate genes related to sheep gastrointestinal nematode resistance were obtained from the included literature. We searched and downloaded 41 annotated genes from QTL regions related to sheep gastrointestinal nematode resistance from the Sheep QTLdb database (Table S5), which served as “trained genes” for the reference training set in ToppGene gene prioritization analysis. After removing duplicates between the 1580 candidate genes obtained in this study and the trained genes, 1562 genes remained as “test genes” for ToppGene gene prioritization analysis. FDR values were calculated based on ToppGene’s overall p-values, and the top 75 genes with highest priority were selected using FDR ≤ 0.05 for subsequent analysis (Table S6). Among the 75 high-priority genes, 18 are derived from highly credible candidate genes, 57 are from moderately credible candidate genes, 40 genes are from parasitological trait-related genes, 11 genes are from physiological trait-related genes, and 26 genes are from physiological trait-related genes. Among all high-priority genes, only 9 genes have an FDR < 0.05 (TNF, STAT3, STAT5A, PDGFB, ADRB2, MAPT, ITGB3, SMO, GH1) and therefore are identified as core genes.

Based on our candidate genes’ confidence levels and trait types, we performed sensitivity analyses between different gene clusters. The results of analysis of high-confidence genes only show that the top 18 genes perfectly overlap with the high-priority genes. The analysis results of middle-confidence genes only show that the top 47 genes perfectly overlap with the high-priority genes, and all 57 initially determined high-priority genes are still within the top 73 rankings. The analysis results of parasitological genes only show that the top 33 genes perfectly overlap with the high-priority genes, and all 40 initially determined high-priority genes are still within the top 48 rankings. The analysis results of physiological genes only show that the top 23 genes perfectly overlap with the high-priority genes, and all 27 initially determined high-priority genes are still within the top 52 rankings. The analysis results of immunological genes only show that the top 7 genes perfectly overlap with the high-priority genes, and all 11 initially determined high-priority genes are still within the top 16 rankings.

### 3.4. Functional Enrichment Analysis and Gene Network Analysis

The 75 high-priority candidate genes were significantly enriched in 13 GO BP terms, 1 GO CC term and 9 GO MF terms (Table S7). GO BP terms were mainly related to response to stimulus, while signaling GO CC terms were mainly enriched in cytoskeleton. GO MF terms were mainly enriched in protein kinase activity and acetyl-CoA carboxylase activity. KEGG pathway analysis showed significant enrichment in 26 pathways (Table S7), with the top 5 most enriched pathways being the JAK-STAT signaling pathway, pathways in cancer, inflammatory bowel disease, Th17 cell differentiation, and Hepatitis B.

## 4. Discussion

### 4.1. Main Findings and Significance of the Systematic Review

Through systematic review of 22 GWASs, this research successfully identified and integrated 1580 candidate genes related to gastrointestinal nematode resistance from large-scale data encompassing 28,033 samples across 32 sheep breeds/populations worldwide. Gene prioritization analysis enabled us to screen 75 high-confidence candidate genes and identify 9 key core genes (TNF, STAT3, STAT5A, PDGFB, ADRB2, MAPT, ITGB3, SMO, GH1). These findings provide important scientific evidence for understanding the genetic architecture of parasite resistance traits in sheep.

The included studies showed a broad geographic distribution, covering 11 countries, with more studies from Brazil (27.3%) and the United States (18.2%), reflecting the widespread nature of sheep parasite infections across different geographic environments and climate conditions. The high breed diversity involved, including adaptive indigenous breeds (such as Red Maasai, Santa Inês, Morada Nova) and improved commercial breeds (such as Merino, Romney), provides rich material for understanding genetic differences between breeds [43,51].

Our systematic review covered 32 different sheep breeds/populations, providing valuable opportunities to compare the genetic architecture of parasite resistance between different breeds. Research shows significant genetic differences between breeds, particularly between indigenous and improved breeds [51,54,55]. For example, traditionally managed indigenous sheep have evolved various strategies to enhance GIN resistance through long-term natural selection processes. Whole-genome studies of Tunisian indigenous sheep revealed multiple genomic regions associated with parasite resistance that are often not significant in improved breeds [28]. This suggests that breeding practices should make full use of beneficial genetic resources from indigenous breeds.

From our included studies, we can see that GWAS technology application in sheep gastrointestinal nematode resistance research continues to develop and improve. Currently, related research mainly still uses 50 K SNP chips, with recent studies beginning to apply 600 K high-density chips and even imputation strategies using whole-genome sequencing data [28,38,48,50]. Particularly noteworthy is that copy number variations (CNVs), as an important class of structural variations, are beginning to receive attention for their role in parasite resistance. Estrada-Reyes et al. (2022) first applied CNV analysis in sheep parasite resistance GWAS research, discovering 14 CNV regions related to resistance [41]. This provides a new perspective for understanding the genetic mechanisms of parasite resistance.

### 4.2. Mechanistic Interpretation of Core Resistance Genes and Pathways

TNF represents a critical inflammatory mediator in sheep nematode resistance. Estrada-Reyes et al. identified TNF pathway components among significantly associated loci in Florida Native sheep resistant to *Haemonchus contortus*, suggesting direct involvement in resistance mechanisms [52]. In sheep, TNF coordinates early inflammatory responses that facilitate immune cell recruitment to parasitized tissues and enhance antigen presentation efficiency. This cytokine also regulates endothelial activation and vascular permeability, enabling effective immune surveillance of gastrointestinal mucosa during nematode infection.

Our functional enrichment analysis results show that the JAK-STAT signaling pathway is one of the most important regulatory pathways in sheep anti-parasite immune responses. This pathway ranked in the top five in enrichment analysis, and several core genes identified in network analysis are related to this pathway, including STAT3, STAT5, PDGFB and GH1. The JAK-STAT pathway is a classic route for cytokine signal transduction, playing key roles in both innate and adaptive immunity. STAT3, as the core transcription factor of the JAK-STAT pathway, not only participates in inflammatory response regulation but also plays important roles in immune cell differentiation and cytokine production [56]. Al Kalaldeh et al. detected genomic regions containing STAT3 and related signaling components associated with resistance to gastrointestinal nematodes in Australian sheep populations and with STAT3 colocalization with immune-related CNV regions [43]. Polymorphisms within the STAT3 gene are associated with FEC in Florida Native sheep [52]. STAT5A complements STAT3 function in orchestrating protective immunity against gastrointestinal nematodes. Carracelas et al. found that STAT5 pathway enrichment in genomic regions is associated with parasite resistance in Corriedale sheep [42]. STAT5A mediates IL-4 and IL-13 signaling, critical for eosinophil activation, mast cell degranulation, and tissue repair following parasite-induced damage. This protein also regulates memory T-cell development, contributing to enhanced secondary responses upon reinfection. PDGFB is an important member of the platelet-derived growth factor (PDGF) family. Currently, PDGFB is generally not considered a direct anti-parasitic molecule, but it plays an important supportive role in maintaining host defense mechanisms and tissue integrity, and its expression and function are indeed affected by parasitic infections. During malaria infection, extracellular products of Plasmodium falciparum affect endothelial cell integrity, including abnormal regulation of the PDGF-BB (PDGFB) signaling pathway [56]. Toxoplasma infection impairs PDGF signaling in cardiomyocytes, with reduced detection signals for both PDGF-AA and PDGF-AB/PDGF-BB in infected cells [57]. GH1 encodes growth hormones and is an important gene regulating animal growth, development, and metabolism. Research shows that there is a complex relationship between GH1 gene expression and parasite resistance in sheep. In gene expression studies related to nematode resistance in ruminants, GH1 was identified as one of the candidate genes [58]. Transgenic studies found that sheep carrying growth hormone transgenes grow faster but have a heavier parasite burden [59]. Different sheep breeds also show differences in polymorphisms of the GH1 gene [60].

Our GO enrichment analysis shows that response to stimulus is the most significantly enriched biological process, which is highly consistent with host immune response mechanisms triggered by parasite infections, in which the core genes TNF, STAT3, STAT5A, PDGFB, ADRB2 and SMO were included. The ADRB2 gene is an important member of the G protein-coupled receptor superfamily. In genome-wide selection scans of sheep, ADRB2 was identified as a gene associated with parasite resistance [61], and its involvement in regulating β-adrenergic receptor signaling has been shown to suppress various immune responses, including autoimmune diseases and infection models [62]. It was also reported as a candidate gene in the anti-parasitic genome-wide association studies included in this study [46]. SMO plays a key signal transduction role in the Hedgehog signaling pathway [63], participating in the control of intestinal epithelial cell proliferation and differentiation, as well as regulating barrier function and immune homeostasis [64,65]. This suggests that SMO-mediated hedgehog signaling may be an important mechanism for maintaining intestinal resistance to parasite invasion. The SMO gene was identified as a candidate gene associated with gastrointestinal nematode resistance traits in a single-step genome-wide association study with weighted SNPs on Santa Ines sheep included in this review [39].

### 4.3. Research Prospects and Challenges

Based on our identified high-priority candidate genes, targeted molecular markers can be developed for marker-assisted selection (MAS). SNP loci significantly associated across multiple breeds and environments particularly have high application value [43,45]. Therefore, we recommend prioritizing development of functional markers related to core genes discovered in this study (such as TNF, STAT3, STAT5A, etc.).

Our restriction to PubMed and Web of Science, while providing high-quality indexed studies, may have missed relevant studies in agricultural databases (CAB Abstracts, AGRICOLA) or regional publications. The English-only restriction likely underrepresented research from major sheep-producing regions where results might be published in local languages. Future reviews should consider broader database coverage and multilingual searches.

In actual breeding, it is necessary to balance relationships between parasite resistance and other important economic traits (such as growth performance, reproductive performance, wool production performance, etc.). Constructing multi-trait selection indices including anti-parasite traits will help achieve comprehensive optimization of breeding objectives [11]. Genomic selection provides better tools for simultaneous multi-trait improvement. By constructing multi-trait genomic-estimated breeding values (multi-trait GEBVs), parasite resistance can be improved while minimizing negative impacts on other important traits [66]. Personalized precision breeding strategies should be developed for different breeds and environmental conditions. For indigenous breeds that already have certain resistance foundations, the focus should be on utilizing their advantageous genetic resources and further improving resistance levels through genomic selection [15,17]. For improved breeds with weaker resistance, resistance genes can be introduced through crossbreeding and gene introgression.

It should be noted that most of our identified candidate genes still lack adequate functional validation. Although bioinformatics-based functional predictions provide important clues, the specific roles of these genes in parasite resistance still require validation through in vitro and in vivo experiments. Future research should focus on (1) validating the functions of key candidate genes through gene knockout/knock-in experiments; (2) using transcriptome sequencing technology to analyze dynamic changes in gene expression during parasite infection; and (3) revealing the complete picture of molecular mechanisms through proteomics and metabolomics studies.

Furthermore, parasite resistance, as a complex trait, is easily influenced by environmental factors, including climate conditions, nutrition levels, and management practices [67,68]. Genotype × environment interaction effects (G × E) may significantly affect the stability and universality of GWAS results. Future research needs to validate candidate gene effects under more diverse environmental conditions and explore how to use G × E interaction information to improve breeding selection efficiency. Meanwhile, meta-analysis of multi-environment trials will help identify core genes that are stably expressed under different environmental conditions.

## 5. Conclusions

This study provides important insights for understanding the genetic architecture of gastrointestinal nematode resistance in sheep through a large-scale systematic review and gene prioritization analysis. The 75 high-priority candidate genes and 11 core genes we identified, particularly genes related to the JAK-STAT signaling pathway, lay the foundation for deeper understanding of the molecular mechanisms of parasite resistance. These findings not only advance the understanding of the genetic basis of complex resistance traits but also provide scientific evidence for developing efficient genomic selection strategies. Future research should focus on functional validation of candidate genes, multi-environment validation, and translation to practical breeding applications, to achieve effective transfer of scientific achievements to production practice. Through continuous genetic improvement, the ultimate goal is to develop sheep breeds that possess both excellent production performance and strong parasite resistance, contributing to the sustainable development of the global sheep industry.

## Figures and Tables

**Table 1:** Incorporated quality assessment and bias risk evaluation scoring criteria for the literature.

Evaluation Indicators	0 Points	1 Points	2 Points	3 Points
Population Structure Control	No population stratification	Either PCA or genomic control applied	Principal component analysis + genomic control	-
Sample Size Adequacy	<200 animals	200–500 animals	>500 animals	-
Phenotype Definition and Measurement	Poorly defined or non-standardized phenotypes	Standardized phenotyping protocols	-	-
Genotyping Platform and Coverage	<20 K SNPs	20–50 K SNPs	50 K SNPs or whole-genome sequencing	-
Quality Control Procedures	Minimal or unclear QC procedures	Basic QC applied	Comprehensive QC with MAF, HWE, call rate, sample filtering	-
Multiple Testing Correction	No multiple testing correction	Bonferroni or FDR correction applied	-	-
Replication and Validation	In single breeds	In multiple breeds	-	-
Reference Genome	Version information unclear	Cannot be used for lift over conversion	Can be used for lift over conversion	-
Data Reproducibility	Limited availability	Difficult to re-annotate locus information or only genomic regions available	Partial locus and candidate genes extractable	Detailed and extractable locus and candidate gene information

**Table 2:** Source of data, sample size, country and breed for 23 studies used for this analysis.

Source Study	Sample Size	Country	Breed
Arzik, Y. et al., 2022 [35]	475	Turkey	Akkaraman
Niciura, S.C.M. et al., 2022 [36]	44	Brazil	Morada Nova
Thorne, J.W. et al., 2023 [37]	329	United States	Rambouillet, Dorper, White Dorper
Becker, G.M. et al., 2022 [38]	583	United States	Katahdin
Berton, M.P. et al., 2022 [39]	576	Brazil	Santa Inês
Carracelas, B. et al., 2022 [40]	375	Uruguay	Corriedale
Estrada-Reyes, Z.M. et al., 2022 [41]	261	United States	Florida Native
Ahbara, A.M. et al., 2021 [28]	92	Tunisia	Tunisian indigenous sheep
Vera, B. et al., 2024 [42]	1697	Uruguay	Australian Merino
Al Kalaldeh, M. et al., 2019 [43]	7539	Australia	Merino, Poll Dorset, Suffolk, White Suffolk, White Dorper, Border Leicester, and crossed
Yaman, Y. et al., 2024 [44]	48	Brazil	Morada Nova sheep
Pickering, N.K. et al., 2015 [45]	8705	New Zealand	Romney, Coopworth, Perendale, Texel, CompRCP, CompRCPT, CompCRP
Stafuzza, N.B. et al., 2023 [46]	589	Brazil	Santa Ines
Atlija, M. et al., 2016 [47]	518	Spain	Spanish Churra
Al Kalaldeh, M. et al., 2019 [48]	1881	Australia	Merino, Poll Dorset, Border Leicester, White Suffolk, and other
Persichilli, C. et al., 2025 [26]	120	Italy	Comisana, Massese
Costa, K.A. et al., 2025 [49]	638	Brazil	Santa Ines
Pacheco, A. et al., 2024 [50]	1766	United Kingdom (Scotland)	Scottish Blackface
Benavides, M.V. et al., 2015 [51]	371	Kenya	Red Maasai × Dorper double backcross group
Estrada-Reyes, Z.M. et al., 2019 [52]	100	United States	Florida Native
Riggio, V. et al., 2013 [25]	752	United Kingdom	Scottish Blackface
Berton, M.P. et al., 2017 [53]	574	Brazil	Santa Inês

## Data Availability

The original contributions presented in this review are included in the article within the text; further inquiries can be directed to the corresponding author.

## Supplementary Materials

The following supporting information can be downloaded at https://www.mdpi.com/article/10.3390/genes16101151/s1, Table S1. Specific information and quality assessment and bias risk evaluation scores of 22 included studies. Table S2. SNP and CNV locus information extracted from the included study and lift over conversion results. Table S3. Genomic region information extracted from the included study and lift over conversion results. Table S4. List of high and middle confidence candidate genes. Table S5. Sheep parasite resistance trait related genes from SheepQTLdb. Table S6. List of 75 high-priority candidate genes obtained based on ToppGene gene prioritization analysis. Table S7. GO terms and KEGG pathways significantly enriched by high-priority genes. Table S8. 75 high priority candidate gene location information and sources. Table S9. genes-16-01151-PRISMA_2020_checklist.

## References

[1] Bishop S.C., Stear M.J. (2003). Modeling of host genetics and resistance to infectious diseases: Understanding and controlling nematode infections. Vet. Parasitol..

[2] Charlier J., van der Voort M., Kenyon F., Skuce P., Vercruysse J. (2014). Chasing helminths and their economic impact on farmed ruminants. Trends Parasitol..

[3] Roeber F., Jex A.R., Gasser R.B. (2013). Impact of gastrointestinal parasitic nematodes of sheep, and the role of advanced molecular tools for exploring epidemiology and drug resistance—An Australian perspective. Parasit. Vectors.

[4] Hayward A.D. (2022). Genetic parameters for resistance to gastrointestinal nematodes in sheep: A meta-analysis. Int. J. Parasitol..

[5] Coltman D.W., Wilson K., Pilkington J.G., Stear M.J., Pemberton J.M. (2001). A microsatellite polymorphism in the gamma interferon gene is associated with resistance to gastrointestinal nematodes in a naturally-parasitized population of Soay sheep. Parasitology.

[6] Kaplan R.M. (2004). Drug resistance in nematodes of veterinary importance: A status report. Trends Parasitol..

[7] Kotze A.C., Prichard R.K. (2016). Anthelmintic resistance in *Haemonchus contortus*: History, mechanisms and diagnosis. Adv. Parasitol..

[8] Jackson F., Coop R.L. (2000). The development of anthelmintic resistance in sheep nematodes. Parasitology.

[9] Torres-Acosta J.F., Hoste H. (2008). Alternative or improved methods to limit gastro-intestinal parasitism in grazing sheep and goats. Small Rumin. Res..

[10] Bishop S.C., Morris C.A. (2007). Genetics of disease resistance in sheep and goats. Small Rumin. Res..

[11] Brown D.J., Fogarty N.M. (2017). Genetic relationships between internal parasite resistance and production traits in Merino sheep. Anim. Prod. Sci..

[12] Safari E., Fogarty N.M., Gilmour A.R. (2005). A review of genetic parameter estimates for wool, growth, meat and reproduction traits in sheep. Livest. Prod. Sci..

[13] McRae K.M., Stear M.J., Good B., Keane O.M. (2015). The host immune response to gastrointestinal nematode infection in sheep. Parasite Immunol..

[14] Stear M.J., Doligalska M., Donskow-Schmelter K. (2007). Alternatives to anthelmintics for the control of nematodes in livestock. Parasitology.

[15] McManus C., do Prado Paim T., de Melo C.B., Brasil B.S., Paiva S.R. (2014). Selection methods for resistance to and tolerance of helminths in livestock. Parasite.

[16] Baker R.L., Mwamachi D.M., Audho J.O., Thorpe W., Barger I.A. (1999). Resistance of Galla and Small East African goats in the sub-humid tropics to gastrointestinal nematode infections and the peri-parturient rise in faecal egg counts. Vet. Parasitol..

[17] Zvinorova P.I., Halimani T.E., Muchadeyi F.C., Matika O., Riggio V., Dzama K. (2016). Breeding for resistance to gastrointestinal nematodes—The potential in low-input/output small ruminant production systems. Vet. Parasitol..

[18] Woolaston R.R., Baker R.L. (1996). Prospects of breeding small ruminants for resistance to internal parasites. Int. J. Parasitol..

[19] Stear M.J., Strain S., Bishop S.C. (1999). Mechanisms underlying resistance to nematode infection. Int. J. Parasitol..

[20] Goddard M.E., Hayes B.J. (2009). Mapping genes for complex traits in domestic animals and their use in breeding programmes. Nat. Rev. Genet..

[21] Andersson L., Georges M. (2004). Domestic-animal genomics: Deciphering the genetics of complex traits. Nat. Rev. Genet..

[22] Bush W.S., Moore J.H. (2012). Chapter 11: Genome-wide association studies. PLoS Comput. Biol..

[23] Hirschhorn J.N., Daly M.J. (2005). Genome-wide association studies for common diseases and complex traits. Nat. Rev. Genet..

[24] Marshall K., Maddox J.F., Lee S.H., Zhang Y., Kahn L., Graser H.U., Gondro C., Walkden-Brown S.W., van der Werf J.H. (2009). Genetic mapping of quantitative trait loci for resistance to *Haemonchus contortus* in sheep. Anim. Genet..

[25] Riggio V., Matika O., Pong-Wong R., Stear M.J., Bishop S.C. (2013). Genome-wide association and regional heritability mapping to identify loci underlying variation in nematode resistance and body weight in Scottish Blackface lambs. Heredity.

[26] Persichilli C., Biffani S., Senczuk G., Di Civita M., Bitew M.K., Bosco A., Rinaldi L., Grande S., Cringoli G., Pilla F. (2025). A case-control genome-wide association study of estimated breeding values for resistance to gastrointestinal nematodes in two local dairy sheep breeds. Animal.

[27] Vera B., Navajas E.A., Carracelas B., Ciappesoni G. (2025). Accuracy of genomic predictions for resistance to gastrointestinal parasites in Australian Merino sheep in Uruguay. Genes..

[28] Ahbara A.M., Rouatbi M., Rjeibi M.R., Ben Salem I., Rekik M., Haile A., Rischkowsky B., Mwacharo J.M. (2021). Genome-wide insights on gastrointestinal nematode resistance in autochthonous Tunisian sheep. Sci. Rep..

[29] Page M.J., McKenzie J.E., Bossuyt P.M., Boutron I., Hoffmann T.C., Mulrow C.D., Shamseer L., Tetzlaff J.M., Akl E.A., Brennan S.E. (2021). The PRISMA 2020 statement: An updated guideline for reporting systematic reviews. BMJ.

[30] Fu W., Wang R., Nanaei H.A., Wang J., Hu D., Jiang Y. (2022). RGD v2.0: A major update of the ruminant functional and evolutionary genomics database. Nucleic Acids Res..

[31] Hu Z.L., Park C.A., Reecy J.M. (2022). Bringing the Animal QTLdb and CorrDB into the future: Meeting new challenges and providing updated services. Nucleic Acids Res..

[32] Chen J., Bardes E.E., Aronow B.J., Jegga A.G. (2009). ToppGene Suite for gene list enrichment analysis and candidate gene prioritization. Nucleic Acids Res..

[33] Benjamini Y., Hochberg Y. (1995). Controlling the false discovery rate: A practical and powerful approach to multiple testing. J. R. Stat. Soc. Ser. B Stat. Methodol..

[34] Kolberg L., Raudvere U., Kuzmin I., Adler P., Vilo J., Peterson H. (2023). g:Profiler-interoperable web service for functional enrichment analysis and gene identifier mapping (2023 update). Nucleic Acids Res..

[35] Arzik Y., Kizilaslan M., White S.N., Piel L.M.W., Çınar M.U. (2022). Genomic Analysis of Gastrointestinal Parasite Resistance in Akkaraman Sheep. Genes.

[36] Niciura S.C.M., Benavides M.V., Okino C.H., Ibelli A.M.G., Minho A.P., Esteves S.N., Chagas A.C.S. (2022). Genome-Wide Association Study for *Haemonchus contortus* Resistance in Morada Nova Sheep. Pathogens.

[37] Thorne J.W., Redden R., Bowdridge S.A., Becker G.M., Stegemiller M.R., Murdoch B.M. (2023). Genome-Wide Analysis of Sheep Artificially or Naturally Infected with Gastrointestinal Nematodes. Genes.

[38] Becker G.M., Burke J.M., Lewis R.M., Miller J.E., Morgan J.L.M., Rosen B.D., Van Tassell C.P., Notter D.R., Murdoch B.M. (2022). Variants Within Genes EDIL3 and ADGRB3 are Associated with Divergent Fecal Egg Counts in Katahdin Sheep at Weaning. Front. Genet..

[39] Berton M.P., da Silva R.P., Banchero G., Mourão G.B., Ferraz J.B.S., Schenkel F.S., Baldi F. (2022). Genomic integration to identify molecular biomarkers associated with indicator traits of gastrointestinal nematode resistance in sheep. J. Anim. Breed. Genet..

[40] Carracelas B., Navajas E.A., Vera B., Ciappesoni G. (2022). Genome-Wide Association Study of Parasite Resistance to Gastrointestinal Nematodes in Corriedale Sheep. Genes.

[41] Estrada-Reyes Z.M., Ogunade I.M., Pech-Cervantes A.A., Terrill T.H. (2022). Copy number variant-based genome wide association study reveals immune-related genes associated with parasite resistance in a heritage sheep breed from the United States. Parasite Immunol..

[42] Vera B., Navajas E.A., Peraza P., Carracelas B., Van Lier E., Ciappesoni G. (2024). Genomic Regions Associated with Resistance to Gastrointestinal Parasites in Australian Merino Sheep. Genes.

[43] Al Kalaldeh M., Gibson J.P., Lee S.H., Gondro C., van der Werf J.H. (2019). Detection of genomic regions underlying resistance to gastrointestinal parasites in Australian sheep. Genet. Sel. Evol..

[44] Niciura S.C.M., Cardoso T.F., Ibelli A.M.G., Okino C.H., Andrade B.G., Benavides M.V., Chagas A.C.S., Esteves S.N., Minho A.P., Regitano L.C.A. (2024). Multi-omics data elucidate parasite-host-microbiota interactions and resistance to *Haemonchus contortus* in sheep. Parasit. Vectors.

[45] Pickering N.K., Auvray B., Dodds K.G., McEwan J.C. (2015). Genomic prediction and genome-wide association study for dagginess and host internal parasite resistance in New Zealand sheep. BMC Genom..

[46] Stafuzza N.B., de Freitas A.C., Mioto M.B., de Oliveira Silva R.M., de Oliveira Fragomeni B., Pedrosa V.B., da Costa R.L.D., de Paz C.C.P. (2023). Weighted Single-Step Genome-Wide Association Study and Functional Enrichment Analyses for Gastrointestinal Nematode Resistance Traits in Santa Ines Sheep. Vet. Parasitol..

[47] Atlija M., Arranz J.J., Martinez-Valladares M., Gutierrez-Gil B. (2016). Detection and replication of QTL underlying resistance to gastrointestinal nematodes in adult sheep using the ovine 50K SNP array. Genet. Sel. Evol..

[48] Al Kalaldeh M., Gibson J.P., Duijvesteijn N., Daetwyler H.D., MacLeod I., Moghaddar N., Lee S.H., van der Werf J.H.J. (2019). Using imputed whole-genome sequence data to improve the accuracy of genomic prediction for parasite resistance in Australian sheep. Genet. Sel. Evol..

[49] Costa K.A., Araujo A.C., Fonseca P.A.S., Silva H.T., Menegatto L.S., de Freitas L.A., Cardoso C.M., Carvalho Filho I., Otto P.I., Costa R.L.D.D. (2025). Genetic parameters and haplotype-based genome-wide association study of indicator traits for gastrointestinal parasite resistance in Santa Ines sheep. Vet. Parasitol..

[50] Pacheco A., Banos G., Lambe N., McLaren A., McNeilly T.N., Conington J. (2024). Genome-wide association studies of parasite resistance, productivity and immunology traits in Scottish Blackface sheep. Animal.

[51] Benavides M.V., Sonstegard T.S., Kemp S., Mugambi J.M., Gibson J.P., Baker R.L., Hanotte O., Marshall K., Van Tassell C. (2015). Identification of novel loci associated with gastrointestinal parasite resistance in a Red Maasai x Dorper backcross population. PLoS ONE.

[52] Estrada-Reyes Z.M., Rae O., Postley C., Jiménez Medrano M.B., Leal Gutiérrez J.D., Mateescu R.G. (2019). Association study reveals Th17, Treg, and Th2 loci related to resistance to *Haemonchus contortus* in Florida Native sheep. J. Anim. Sci..

[53] Berton M.P., de Oliveira Silva R.M., Peripolli E., Stafuzza N.B., Martin J.F., Álvarez M.S., Gavinã B.V., Toro M.A., Banchero G., Oliveira P.S. (2017). Genomic regions and pathways associated with gastrointestinal parasites resistance in Santa Inês breed adapted to tropical climate. J. Anim. Sci. Biotechnol..

[54] Baker R.L., Mugambi J.M., Audho J.O., Carles A.B., Thorpe W. (2004). Genotype by environment interactions for productivity and resistance to gastro-intestinal nematode parasites in Red Maasai and Dorper sheep. Anim. Sci..

[55] Woolaston R.R., Windon R.G. (2001). Selection of sheep for response to *Trichostrongylus colubriformis* larvae: Genetic parameters. Anim. Sci..

[56] Piatti L., Batzilla A., Nakaki F., Fleckenstein H., Korbmacher F., Long R.K.M., Schraivogel D., Hawkins J.A., Romero-Uruñuela T., López-Gutiérrez B. (2025). Plasmodium falciparum egress disrupts endothelial junctions and activates JAK-STAT signaling in a microvascular 3D blood-brain barrier model. Nat. Commun..

[57] de Castro L.L., Gomes da Rosa B., Peixoto-Rodrigues M.C., Revoredo Vicentino A.R., Fraga-Junior V.d.S., Cascabulho C.M., Diniz L.P., Benjamim C.F., Bracko O., Scharfstein J. (2025). Toxoplasma gondii impairs CX3CL1/fractalkine shedding from mouse cortical neurons, leading to microglia activation. Microbiol. Spectr..

[58] Araujo R.N., Padilha T., Zarlenga D., Sonstegard T., Connor E.E., Van Tassel C., Lima W.S., Nascimento E., Gasbarre L.C. (2019). Use of a candidate gene array to delineate gene expression patterns in cattle selected for resistance or susceptibility to intestinal nematodes. Vet. Parasitol..

[59] Adams N.R., Briegel J.R., Ward K.A. (2002). The impact of a transgene for ovine growth hormone on the performance of two breeds of sheep. J. Anim. Sci..

[60] Becker G.M., Thorne J.W., Burke J.M., Lewis R.M., Notter D.R., Morgan J.L.M., Schauer C.S., Stewart W.C., Redden R.R., Murdoch B.M. (2024). Genetic diversity of United States Rambouillet, Katahdin and Dorper sheep. Genet. Sel. Evol..

[61] Fonseca P.A.S., Suárez-Vega A., Arranz J.J., Gutiérrez-Gil B. (2024). Integration of selective sweeps across the sheep genome: Understanding the relationship between production and adaptation traits. Genet. Sel. Evol..

[62] Qiao G., Chen M., Bucsek M.J., Repasky E.A., Hylander B.L. (2018). Adrenergic Signaling: A Targetable Checkpoint Limiting Development of the Antitumor Immune Response. Front. Immunol..

[63] Arensdorf A.M., Marada S., Ogden S.K. (2016). Smoothened Regulation: A Tale of Two Signals. Trends Pharmacol. Sci..

[64] Kolev H.M., Kaestner K.H. (2023). Mammalian Intestinal Development and Differentiation-The State of the Art. Cell. Mol. Gastroenterol. Hepatol..

[65] Hou Q., Huang J., Ayansola H., Masatoshi H., Zhang B. (2021). Intestinal Stem Cells and Immune Cell Relationships: Potential Therapeutic Targets for Inflammatory Bowel Diseases. Front. Immunol..

[66] Daetwyler H.D., Hickey J.M., Henshall J.M., Dominik S., Gredler B., van der Werf J.H., Hayes B.J. (2010). Accuracy of estimated genomic breeding values for wool and meat traits in a multi-breed sheep population. Anim. Prod. Sci..

[67] Beraldi D., Craig B.H., Bishop S.C., Hopkins J., Pemberton J.M. (2008). Phenotypic analysis of host-parasite interactions in lambs infected with Teladorsagia circumcincta. Int. J. Parasitol..

[68] Sallé G., Doyle S.R., Cortet J., Cabaret J., Berriman M., Holroyd N., Cotton J.A. (2019). The global diversity of *Haemonchus contortus* is shaped by human intervention and climate. Nat. Commun..

